# Big Data-Enabled Analysis of DRGs-Based Payment on Stroke Patients in Jiaozuo, China

**DOI:** 10.1155/2020/6690019

**Published:** 2020-12-02

**Authors:** Dawei Qiao, Yanru Zhang, Ateeq ur Rehman, Mohammad R. Khosravi

**Affiliations:** ^1^School of Medicine, Henan Polytechnic University, Jiaozuo 454000, China; ^2^Computer Science Department, Abdul Wali Khan University Mardan, Mardan, KPK, Pakistan; ^3^Department of Computer Engineering, Persian Gulf University, Bushehr 75168, Iran

## Abstract

Stroke is the first leading cause of mortality in China with annual 2 million deaths. According to the National Health Commission of the People's Republic of China, the annual in-hospital costs for the stroke patients in China reach ¥20.71 billion. Moreover, multivariate stepwise linear regression is a prevalent big data analysis tool employing the statistical significance to determine the explanatory variables. In light of this fact, this paper aims to analyze the pertinent influence factors of diagnosis related groups- (DRGs-) based stroke patients on the in-hospital costs in Jiaozuo city of Henan province, China, to provide the theoretical guidance for medical payment and medical resource allocation in Jiaozuo city of Henan province, China. All medical data records of 3,590 stroke patients were from the First Affiliated Hospital of Henan Polytechnic University between 1 January 2019 and 31 December 2019, which is a Class A tertiary comprehensive hospital in Jiaozuo city. By using the classical statistical and multivariate linear regression analysis of big data related algorithms, this study is conducted to investigate the influence factors of the stroke patients on in-hospital costs, such as age, gender, length of stay (LoS), and outcomes. The essential findings of this paper are shown as follows: (1) age, LoS, and outcomes have significant effects on the in-hospital costs of stroke patients; (2) gender is not a statistically significant influence factor on the in-hospital costs of the stroke patients; (3) DRGs classification of the stroke patients manifests not only a reduced mean LoS but also a peculiar shape of the distribution of LoS.

## 1. Introduction

Stroke is an acute cerebrovascular disease, which is characterized by the sudden numbness of some parts of the body, such as face, arm, or leg. This is a group of diseases caused by brain tissue damage, caused by blood flow to the brain due to blocked blood vessels or sudden rupture of blood vessels in the brain [1]. With the development of society and the change of life style, stroke becomes one of the main causes of disability and death in the world. According to the report of World Health Organization (WHO), stroke is the second leading cause of deaths in the world, accounting for 11.3% of the total deaths, and near 5.8 million people died of stroke [2, 3]. Global Burden of Stroke (GBS) reported that there are three characteristics for mortality rate caused by stroke in the low-income, lower-middle-income, upper-middle-income, and high-income countries [4]. The mortality rate of stroke in low-income countries is about 43 per 100,000 population. In lower-middle-income and high-income countries, the mortality rate of stroke is about 62 per 100,000 population, while the number of deaths in upper-middle-income countries reaches up to 117 per 100,000 population. This is more than twice as high as in low-income countries.

Recently, with the number of stroke patients increasing, in-hospital costs spent on stroke are increasing year by year. In western counties, the expenditure on stroke accounts for 2%–4% of total healthcare expenditure [5]. In 2008, the total expense of stroke patients in the United States reached up to $40.9 billion, accounting for 2.6% of the annual healthcare expenditure. The total annual expenditure of stroke in the United Kingdom was as much as £25.6 billion in 2019 [6]. Excepting institutionalization cost, the overall expenditure of the stroke for European Union (EU) was €45 billion, accounting for 28% of the total expenditure of cardiovascular diseases [7]. The Aggregate National Healthcare expenditure of Brazil for the ischemic stroke was about £326.9 million from 2006 to 2007 [8]. This cost accounted for a large proportion of the overall healthcare expenditure since only 18% of Brazilians had private health insurance. In 2012, the entire economic expenditure of stroke was $5 billion in Australia, and the loss of healthy life and the total burden of disease cost in 2012 was $49.4 billion [9]. In Korea, the total economic expenditure of the stroke was $3.53 billion, of which $1.74 billion and $1.79 billion were direct costs and indirect costs, respectively [10]. The authors in [11] presented a detailed analysis of financial data on the direct in-hospital costs of the stroke treatment in Lebanon.

In China, stroke is the first leading cause of mortality and disability of adults. In recent years, the incidence, morbidity, mortality, and disability-adjusted life years (DALY) of the stroke in China are on the rise, and the disease burden caused by stroke is quite serious [12–17]. The Global Burden of Disease (GBD) showed that the number of the stroke patients in China was 4.03 million in 2016, and the average growth rate was 8.3% in 1997–2016. With the ageing of the society, the acceleration of urbanization process, and the popular unhealthy life style of residents, the incidence and mortality of the stroke are increasing dramatically in China [18]. The incidences of DALY caused by stroke, respectively, accounted for 4.6% and 9.71% of all diseases in the world and in China [19], where the disease burden of stroke was more than twice the global average. In 2014, the total cost of the stroke outpatient and inpatient service in China was ¥20.71 billion, and the average annual growth rate of medical cost of the stroke was 24.96% [20]. Due to the imbalance of the economic level and specialized stroke resources among the areas of China, the prevention and management of the stroke are facing great challenges in Henan province, China. Henan is located in the center of China, whose resident population in 2018 was 96.05 million, accounting for 6.9% of the total population of China [21]. According to the Chinese Stroke Association (CSA), the incidence of the stroke of Henan province in 2012 was 3.22%, which was the highest among all provinces of China [22]. The number of stroke patients was about 1.5 million, and the direct and indirect cost of stroke were ¥10 billion. Jiaozuo is located in the northwest of Henan, whose population was 3.55 million in 2019. The differences between urban and rural areas and the regional imbalance in stroke prevention and treatment in Henan are more significant, which are prominent problems that need to be solved at present. Therefore, it is of profound importance to investigate the costs of stroke in Jiaozuo.  Table 1 presents the incidence and mortality caused by stroke between 25 and 74 years old from 1987 to 1993.  Table 1 reveals that Henan has higher incident and mortality than the average of China.

How to solve the problem of the rapid growth of costs for the stroke has always been a worldwide concern, which has sparked a great deal of research interest. The pioneering work was conducted by Fetter at al. in Yale University, from which diagnosis related groups (DRGs) were originally proposed [24]. DRGs were first used to monitor the quality of medical services in medical institutions [25]. Then, the second generation DRGs have been realized in 1983, which were introduced as the basis of the hospital paying of healthcare systems. It was shown that, due to the application of DRGs in the United States from 1983 to 1990, the proportion of the total medical expenditure of gross domestic product (GDP) decreased from 16–18% to 7–8% [26]. Since then, DRGs have been adopted by the most developed countries as the prospective payment system (PPS) to control in-hospital cost [27–32]. It is manifested that DRGs-based PPS can not only improve the utilization efficiency of medical resources, but also reduce the length of stay (LoS) in hospital and the stroke burden.

In China, it can be divided into three stages for the DRGs [33]: (1) the first stage is exploring; (2) the second stage is piloting; and (3) the last one is completing. The first stage started from 1980s to 2001. In this stage, DRGs were introduced and explored by Beijing and Tianjin to provide a strong financial incentive for the healthcare of public hospitals [34, 35]. The second stage was motivated by new rural cooperative medical system (NCMS) for rural Chinese residents [36] ‘Notice on Piloting the Simplified DRG-PPS' of China's Ministry of Health (CMH) and advanced Beijing-DRGs (BJ-DRGs), where the DRGs-PPS/genuine DRGs have been piloted in some public hospitals of China [37, 38]. In the third stage, CMH has released two issues of ‘2008 Quality Supervision and Management Manual of Simplified DRGs-PPS' of Central People's Government of the People's Republic of China (CPGPRC) and ‘Five Key Reforms on Medicine and Health' to assist the promotion of DRGs across the entire China [39, 40]. In [41], DRGs-based payment has been conducted on a trial basis in the selected hospitals in Shanghai. Therefore, unlike the DRGs from other developed countries, the DGRs currently being adopted by China can be regarded as a transitional version of other counties' DRGs [33].

Henan has the largest population of China, especially the rural population, which is urgent to keep the balance of medical resources between rural and urban. In 2018, Health Commission of Henan Province (HCHP) has issued the ‘Notification on the Key Points of Provincial Medical and Political Work', which stated that quality control of the first page of medical records should be strengthened [42]. Jiaozuo city is in the northwest of Henan province. Its total area is 4,071 square kilometres, and the city has a permanent population of 3.5971 million. In addition, the urbanization ratio has reached 60.94%, and Jiaozuo has just issued to promptly explore DRGs payment systems in 2019 [43].

In addition, with the development of information and computer technologies, massive growth of data is a great challenge for further applications. To solve this problem, big data mining and analyzing of collected data to extract the valuable information have become main tasks. For this aspect, multivariate linear regression analysis has been identified as an effective evaluation way of big data by employing the statistical significance to determine the explanatory variables. Big data has been extensively adopted in many fields, such as information communication, finance, security, energy, and electricity [19–23]. With successful applications of big data in the above fields, it has provided many conditions and experience for its application in the healthcare field.

Motivated by the above discussion, this paper aims to investigate the effects of China Healthcare Security-DRGs (CHS-DRGs)-based hospital payment on the stroke patients in Jiaozuo area of Henan province. In the following, we simply use “DRGs” to refer to “CHS-DRGs”. Some influence factors of in-hospital cost of the stroke patients are analyzed via multivariate linear regression analysis, which is one of big data algorithms [11], such as age, gender, LoS, and outcomes. In addition, contrary to the existing works, we also study the distributions of length of stay (LoS) for DRGs-based stroke patients since they reveal the burden not only for stroke patients, but also for medical resources for hospitals. This also presents an incentive for hospital to reassign the medical resources for different stroke patients. The synthetization indicates that DRGs can save the medical costs, improve medical service quality, and reduce LoS. Finally, we carry out the analysis of the impact of outcomes on the in-hospital cost with boxplots.

## 2. Materials and Methods

### 2.1. Description of Data

This study is conducted based on the data collections of the First Affiliated Hospital of Henan Polytechnic University of Jiaozuo; 3,590 discharged stroke cases from 1 January 2019 to 31 December 2019 were selected. In order to avoid the extreme casemix, four cases are ignored: (1) LoS is smaller than 1; (2) LoS is larger than 60; (3) the in-hospital cost is smaller than 7000; and (4) the in-hospital cost is larger than 300,000. We select the data of 2019 since the Department of Human Resources and Social Security of Henan Province (DHRSSHP) has issued the notification that DRGs have been determined as the payment of Henan public hospitals of DHRSSHP [44]. The extracted data includes admission number, age, gender, occupation, dates and modes of admission and discharge, admission condition, expense, and diagnostics of cerebral infarction (ICD-10 codes: I60–I63). In addition, patients transferred from other hospitals and dying before discharge are excluded in the extracted data.

### 2.2. Statistical Analysis of Data

The objective of this paper is to analyze the effects of pertinent influence factors of DRGs-related stroke patients on the in-hospital cost in Jiaozuo public hospitals of Henan province. As in China Healthcare Security-DRGs (CHS-DRGs) [45], the data of the stroke patients can be divided into four stroke-related groups based on the major diagnosis, LoS, admission mode, type of stroke, and outcomes. According to the above discussion, the number of effective data is 3,590. The characteristics of stroke-related groups in Jiaozuo hospital are illustrated in Table 2. In this study, readmission and priority patients are omitted since the number of patients of this stroke kind is only two. For the purpose of convenience, LQ and UQ represent lower quartile and upper quartile, respectively. The average payment includes cure fee, bunk fee, western medicine fee, Chinese medicine fee, examination fee, emission fee, surgery fee, lab fee, inspection fee, sanitary materials fee, and other fees.

Groups I60–I62 are of the type hemorrhage, while group I63 is of the type ischemic. As shown in Table 2, I61 has the most LQ of LoS, UQ of LoS, and median of LoS, which are, respectively, 12 days, 34 days, and 22 days. Then, LQ of LoS, UQ of LoS, and median of LoS in I62 are 11 days, 29 days, and 21 days, respectively. I63 has the least LQ of LoS, UQ of LoS, and median LoS of 8 days, 17 days, and 14 days. Although I60 has smaller LQ of LoS (9 days), UQ of LoS (24 days), and median LoS (17 days) than those of I61 and I62, it has the most expensive group due to larger treatment fee, western medicine fee, and sanitary materials fee. The expenses of the other three groups are ¥40,481.09, ¥45,414.56, and ¥18,644.21, respectively. Compared with I60, I61, and I62, I63 has a large proportion of outpatient service. The outpatient service and emergency treatment account for 62.27% and 37.73%, respectively.

From Table 2, it is clearly shown that all groups have large proportions of cure and improvement. Compared with other groups, I62 has the largest death rate, about 5.71%, which is about twice larger than that of I61 and about six times that of I63. Finally, as can be seen, the ischemic group has the least in-hospital cost compared to other groups due to the least LoS and largest improvement.

### 2.3. Measurement of LoS

To analyze the effects of DRGs-related stroke on the LoS and in-hospital cost, we investigate the distribution of the LoS of in-hospital stroke patients, which can be divided into one most expensive group (I60), two more expensive groups (I61 and I62), and one least expenditure group (I63). In light of this fact, the distributions of LoS of four groups are presented in the form of histograms to distinguish the empirical distributions of LoS. To this end, the in-hospital patients can be divided into hemorrhage stroke and ischemic stroke. The distributions of LoS of the above two types are illustrated by the histograms, which can distinguish the empirical distribution of LoS of the two types. The effects of age, gender, and LoS on the cure, improvement, and death for the two groups are analyzed. It is noted that the other terms are removed since they include many kinds of cases.

### 2.4. Measurement of Outcomes

For the purpose of evaluating the performance of the hospital treating of the stroke patients, four outcomes are considered, namely, cure, improvement, unhealed, and death. The above cases are computed on the basis of discharge mode. Moreover, we investigate the distributions of cure, improvement, unhealed, death, and others according to the type of the stroke (hemorrhage and ischemic), as shown in Table 3. This comparison is adopted for the following two reasons: (1) stroke is classified into hemorrhagic and ischemic, which can keep representative datasets; (2) some sampling errors can be controlled by avoiding the cases of small number of stroke patients. Therefore, unhealed of these two groups is removed due to very small number of the stroke patients. The variation, the central tendency, and the outliers for the outcomes of the stroke patients are evaluated by utilizing boxplots. On this basis, the variation is characterized by the first quartile and the third quartile, while the central tendency is measured by the median. The outliers capture the data points outside the boxplot whiskers.

## 3. Results

In this section, we will explore the influence factors of hospitalization expenses of the stroke patients. First, considering age, gender, LoS, and outcome as dependent variables, we study the effects of DRGs of stroke patients on the hospitalization expense via multivariate linear regression analysis. Then, the distributions of LoS of DRGs-related stroke patients for I60, I61, I62, and I63 are discussed. Finally, we present the effects of outcomes of the stroke patients on the hospitalization expenses.

### 3.1. Stroke-Related DRGs

In order to obtain more insights, by utilizing multivariate linear regression analysis [46], we carry out the analysis of influence effects on the total in-hospital costs of the DRGs stroke patients as shown in Table 4. In this table, we take total in-hospital costs as the independent variable and the patient's age, gender, LoS, and outcomes as the dependent variables. Using multivariate linear regression analysis, we can conclude that although the unstandardized coefficient of gender is not statistically significant, the multiple linear regression model has a good agreement with *R* = 0.539, *R*^2^ = 0.291, and ∆*R* = 0.291 due to a *P* value less than 0.05. In addition, the unstandardized coefficients of age, LoS, and outcomes are statistically significant.

### 3.2. Analysis of the Distribution of LoS

**Figure 1:**
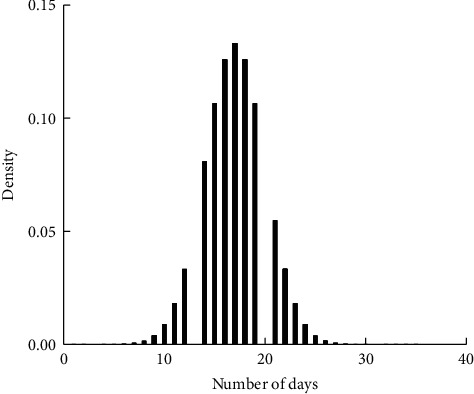
Effects of DRGs-related stroke on the LoS for I60.

**Figure 2:**
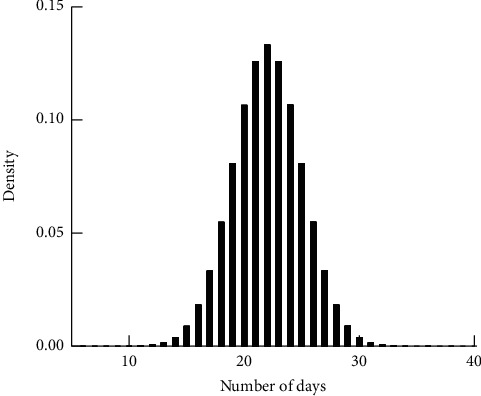
Effects of DRGs-related stroke on the LoS for I61.

**Figure 3:**
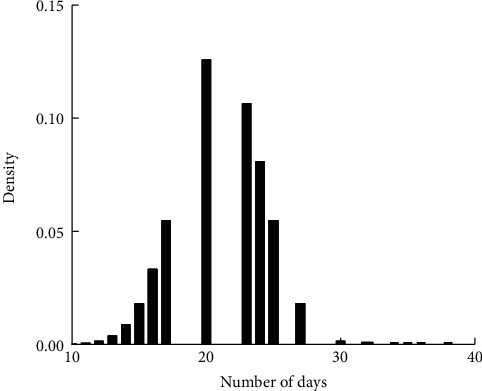
Effects of DRGs-related stroke on the LoS for I62.

**Figure 4:**
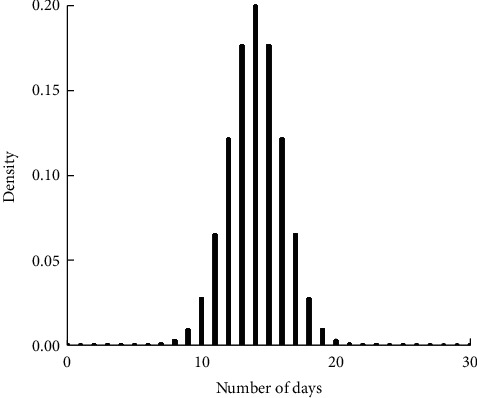
Effects of DRGs-related stroke on the LoS for I63.

As in Figures 1–4, the mean LoS of DRGs-related stroke inpatients for I60, I61, I62, and I63, based on the stroke patient data from the First Affiliated Hospital of Henan Polytechnic University, is 17, 22, 21, and 14 days, respectively. In Figures 1–4, we show the histograms of density of LoS for different groups according to DRGs. From Figures 1 and 3, we can find that there are some vacancies due to the small number of the stroke patients. The histograms in Figures 1–3 almost have the same peak of LoS, which is 13.30%, 13.30%, 12.58%, respectively, while the density of Figure 4 is about 19.95%. This means that ischemic stroke patients have higher peak of LoS than the other three groups. Finally, we can also conclude that DRGs classification of the stroke patients, using LoS as a grouping variable, manifests not only a reduced mean LoS but also a peculiar shape of the distribution of LoS. It happens that most stroke patients stay in hospital for all the groups between 10 and 20 days.

### 3.3. Analysis of Outcomes on Hospitalization Expenses

In this subsection, the boxplots are provided to demonstrate the impacts of outcomes on the in-hospital cost. For analytical accuracy, we remove other terms since they include different influence factors. In addition, the values of hospitalization expenses larger than 200,000 and smaller than 7,000 are removed.

**Figure 5:**
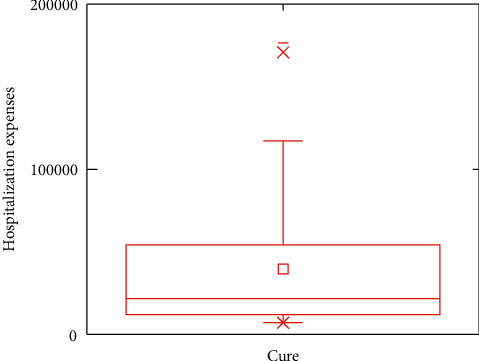
Effects of DRGs-related stroke on the in-hospital cost for cure.

**Figure 6:**
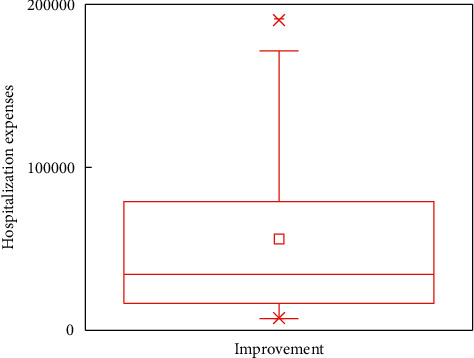
Effects of DRGs-related stroke on the in-hospital cost for improvement.

**Figure 7:**
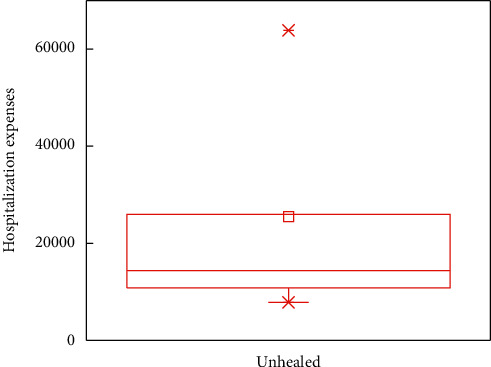
Effects of DRGs-related stroke on the in-hospital cost for unhealed.

**Figure 8:**
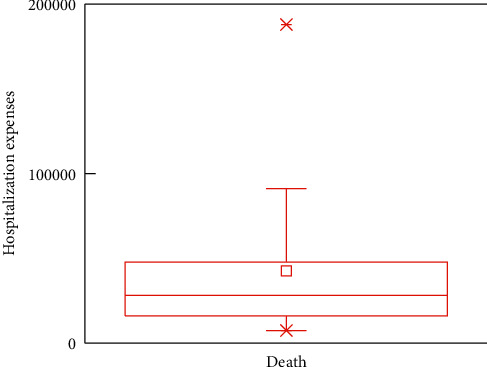
Effects of DRGs-related stroke on the in-hospital cost for death.

Figures 5–8 show boxplots of the hospitalization expenses versus different outcomes, namely, cure, improvement, unhealed, and death. As shown in Figures 5–8, the means of cure and improvement are ¥39,565.73 and ¥56,059.41 compared with ¥25,513.28 and ¥42,597.81 of unhealed and death. The interquartile intervals of cure, improvement, unhealed, and death are [¥11,942.11; ¥54,103.61], [¥16,686.12; ¥76,446.23], [¥16,686.12; ¥25,964.6], and [¥16,124.41; ¥47,837.57], respectively. This means that interquartile intervals of unhealed are the smallest. The differences of others are not very large and concentrate between ¥10,000 and ¥60,000.

## 4. Discussion

Recently, the rapid increase of in-hospital cost has become a common problem in our country. Stroke has become the first leading cause of death among urban and rural residents in China. It not only leads to the loss of labor ability, but also dramatically increases the cost of diagnostic hospitalization. Its burden of disease puts enormous economic pressure on families and society. In addition, payment systems have significant effects on treatment behaviors for stroke patients and hospitals. DRGs are effective ways to improve quality of service and reduce unnecessary medical resources, which has been identified as an important direction for China's medical reform, and DRGs have been adopted as the main payment system [46]. Therefore, it is of great significance to analyze the influencing factors of stroke hospitalization expenses for social and economic benefits.

Based on the multivariate linear regression analysis, we show that stroke is a common cardiovascular disease in Jiaozuo area. Among all the influence factors, gender is the least one, which has no effect on the hospitalization expense. In the future hospital payment system, gender should not be included in group pricing of the payment standard. Moreover, age, LoS, and outcomes are the three significant influence factors that should be included in the payment standard. Finally, we can conclude that the DRGs yield perfect coefficient of variation (CV) and reduction in variation (RIV) results for the medical expense control, which is consistent with the existing literature [47]. Motivated by this, DRGs are the main directions of Jiaozuo's medical reform.

In general, LoS can be classified into value-adding patient days and non-value-adding patient days [48]. The value-adding patient days refer to the days that are meaningful to the diagnosis and prognosis of patients and that patients or payers of medical expenses are willing to pay their expense. Non-value-adding patient days refer to those days that are not necessary, just increasing the cost of hospitalization, and are meaningless to patient's diagnosis. Thus, LoS is an important influence factor of hospitalization expenses of the stroke patients. Note that throughout this paper we use LoS to refer to value-adding patient days. By optimizing medical source allocation and improving medical service, LoS can be shortened and then hospitalization expense can be reduced. LoS of most stroke patients is between 10 and 30 days, and the peaks of density of LoS for hemorrhage and ischemic stroke are lower than 14% and 20%.

Among influence factors, outcomes have significant impacts on the hospitalization expenses, among which improvement and unhealed are the most important ones. This is because improvement has large interquartile intervals and unhealed has the largest mean. Therefore, it is of great significance to analyze the impact of outcomes on hospitalization expenses in Jiaozuo area of Henan province.

## 5. Conclusion and Future Work

The economic conditions and level of development of China do not meet the regulatory requirements for full implementation of DRGs. The improved DRGs schemes have been explored in some cities of China, and they will be gradually piloted across most provinces of China. Jiaozuo of Henan province, as a pilot province of DRGs, has made a lot of important explorations. Under the conditions of the existing resources, however, beginning to screen for common and frequent diseases and investigating the related diseases in each disease group and the key factors for these diseases affecting the medical expenses, according to the standard-setting process simplified based on standardization of clinical path and the accurate cost accounting, will greatly improve the execution efficiency of related diagnosis of Jiaozuo in Henan province.

Utilizing big data related algorithm, this work analyzes the influence factors of DRGs-based stroke patients on in-hospital costs via DRGs; however, how to design a DRGs model for the payment of Jiaozuo hospital will be our further work. In addition, aiming to further enhance the accuracy of the analysis, some advanced machine learning algorithms should be involved, such as decision tree, neural network, and support vector machine. Our analysis methods can be extended to other chronic diseases (hypertension, coronary heart disease, cancer, and diabetes), which are set aside as our future work.

## Figures and Tables

**Table 1:** Summary of average age-standardized incident and mortality of acute stroke in 25–74-year-old population in 1987–1993^a^ [23].

Monitored area	Average monitored population		Incident (/0.1 million)		Mortality (/0.1 million)		Fatality rate (%)	
	Male	Female	Male	Female	Male	Female	Male	Female
Heilongjiang	293, 929	284, 955	646	368	129	89	20	24
Jilin^b^	193, 436	179, 135	508	256	104	68	20	26
Guangdong^b^	37, 780	32, 880	330	167	94	44	28	26
Liaoning	248, 140	243, 237	276	137	113	68	41	49
Beijing	234, 776	241, 248	247	196	91	72	33	36
Henan^b^	65, 005	63, 403	254	191	140	105	55	54
Hebei	60, 673	61, 413	236	166	101	81	43	48
Inner Mongolia^b^	88, 629	86, 754	217	169	77	58	25	34
Shandong^b^	58, 922	52, 374	210	134	65	59	31	44
Fujian^b^	29, 708	28, 905	174	71	112	43	64	60
Xinjiang^b^	17, 898	16, 437	174	198	41	51	23	26
Shanghai	124, 014	133, 591	150	117	72	53	48	45
Szechwan	68, 089	68, 234	133	80	72	46	54	57
Jiangxi^b^	58, 618	54, 853	102	74	46	32	45	43
Jiangsu	112, 749	114, 785	95	55	50	33	52	60
Anhui	38, 107	36, 843	63	45	43	30	68	66
Total	1,730, 473	1, 699, 047	270	161	89	61	33	38

^a^The age-standardized rate is the standardized rate of the world population. ^b^The marked cooperative province in 1987–1989 (Shandong, Fujian, Jiangxi, Henan, and Guangdong) or 1987–1991 (Inner Mongolia).

**Table 2:** Characteristics of stroke-related groups in Jiaozuo hospital (2019)

Code	LQ LoS	UQ LoS	Type of the stroke	Admission mode (%)	Median LoS	Average payment
I60	9 days	24 days	Hemorrhage	Outpatient service (23.33), emergency treatment (76.67)	17 days	¥110,022.70
I61	12 days	34 days	Hemorrhage	Outpatient service (36.20), emergency treatment (63.80)	22 days	¥40,481.09
I62	11 days	29 days	Hemorrhage	Outpatient service (30.56), emergency treatment (69.44)	21 days	¥45,414.65
I63	8 days	17 days	Ischemic	Outpatient service (62.27), emergency treatment (37.73)	14 days	¥18,644.21

**Table 3:** Number and proportion of the outcomes of the stroke patients (2019)

Code	Cure (%)	Improvement (%)	Unhealed (%)	Death (%)	Others (%)	Number of the stroke patients (%)
*G*_1_	124 (32.98)	161 (42.82)	2 (0.53)	10 (21.01)	79 (21.67)	376 (10.47)
*G*_1_	869 (27.04)	2,221 (69.10)	8 (0.25)	29 (0.90)	87 (2.71)	3,214 (89.53)

**Table 4:** Analysis of regression model of the stroke patients and impact factors^c^.

Model	Unstandardized coefficients		Standardized coefficients	*T*	*P*	Collinearity statistics	
	*B*	Std. Error	Beta			Tolerance	VIF
(Constant)	−5,524.606	2,984.944		−1.851	0.064		
Age	−176.765	40.343	−0.062	−4.382	0.000	0.989	1.012
Gender	−961.111	1,079.232	−0.013	−0.891	0.373	0.990	1.010
LoS	1,448.955	40.034	0.513	36.193	0.000	0.984	1.016
Outcomes	9,159.222	571.752	0.227	16.020	0.000	0.983	1.017

^c^ *R* = 0.539, *R*^2^ = 0.291, adjusted *R*^2^ = 0.290, *F* variation = 367.897, Δ*R* = 0.291.

## Data Availability

The data used to support this study are available from the corresponding author upon request.
